# Responding to Key Process Markers as a Focus of Psychotherapy Training and Practice

**DOI:** 10.32872/cpe.11967

**Published:** 2024-04-26

**Authors:** James F. Boswell, Michael J. Constantino, Averi N. Gaines, Ashleigh E. Smith

**Affiliations:** 1 Department of Psychology, University at Albany, State University of New York, Albany, NY, USA; 2 Department of Psychological and Brain Sciences, University of Massachusetts, Amherst, MA, USA; Department of Psychology, University of Trier, Trier, Germany

**Keywords:** psychotherapy training, therapist development, responsiveness, context-responsive psychotherapy integration, evidence-based practice

## Abstract

Historically, evidence-based psychotherapy training has favored the standardized application of discrete treatment packages, with key outcomes being the therapist’s adherence to and competent delivery of theory-prescribed ingredients. However, this model often fails to align with the priorities and values of clinicians, and research casts doubt on the notion that a therapist’s faithful application of treatment protocols is a valid index of clinical expertise. Considering this, training and practice models that emphasize evidence-based clinician flexibility and patient-centered tailoring of interventions are receiving increased attention. In this article, we outline one such model informed by the context-responsive psychotherapy integration (CRPI) framework. Consistent with CRPI principles, we describe several “if this/then try that” marker-response sequences that could become a centerpiece of a more nuanced, clinically representative, and evidence-based psychotherapy training paradigm. Finally, we offer several recommendations for future work on CRPI.

## Highlights

- Psychotherapy training has traditionally valued therapist fidelity to narrow intervention packages over other facilitative therapy processes.
- Growing research supports that psychotherapy training and practice can be more effective if it is personalized to both the patient and provider with contextual and intentional responsivity.

Psychotherapy training, particularly in clinical psychology, has traditionally focused on the theory and application of discrete treatment models. For example, students in a given training program may be expected to complete coursework and supervised practica centered on empirically supported treatments (ESTs), such as cognitive behavioral therapy (CBT) or psychodynamic psychotherapy. Within the CBT tradition, in particular, clinical training has privileged the use of diagnosis-specific treatment protocols or manuals that outline a specific and structured sequence of interventions that, ostensibly, help standardize across patients the clinician actions presumed to be therapeutic. Yet, beyond the training context, the chasm between applied clinical science (which often touts using ESTs like CBT) and everyday clinical practice remains wide.

When clinical researchers lament this science-practice gap, discussions often focus on the perceived low rate at which therapists adopt EST manuals, or even when adopted, therapist variability in adhering to and competently delivering the theory-specific ingredients to their patients. At a basic level, the ability to skillfully apply what one is trained to do strikes us as an appropriate goal for clinical training and practice. However, when considering the psychotherapy research base, it is hard to ignore that greater therapist protocol adherence tends not to correlate with better patient outcomes (Southam-Gerow et al., 2021; Webb et al., 2010). Although there is more evidence to support a small competence-outcome correlation, this finding is far from consistent (Power et al., 2022).

Perhaps the null or mixed results for the adherence- and competence-outcome associations, respectively, are unsurprising when considering the methodological and clinical complexities. For example, despite each construct’s inherent focus on theory-specific treatment actions, competence assessments often include more general therapist behaviors, such as rapport building, which are not necessarily tied to the unique treatment model being delivered. In addition, training therapists to become sustainably adherent and competent in the delivery of a multi-component treatment protocol has proven quite difficult (Frank et al., 2020). Even in controlled trials that involve intensive training and ongoing supervision, treatment adherence and competence can vary significantly among therapists (e.g., Imel et al., 2011). Thus, outside of controlled efficacy studies, adherence and competence levels among routine practicing therapists are predictably even more suspect. Finally, on a broader scale, psychotherapy training practices and trainee outcomes are not consistently linked with *patient* outcomes (Knox & Hill, 2021).

Although we are painting a sobering picture of prevailing training and clinical practices, alternative (or complementary) approaches exist and are receiving more attention (e.g., Boswell et al., 2020). In this article, we (a) introduce and briefly summarize one such approach that veers away from the goals of unwavering adherence to a theory-specific treatment package; (b) suggest potential training structures and activities to support the implementation of this more flexible and context-responsive approach; and (c) provide recommendations for future work in this area.

## Responsive Clinical Practice and Training

It is important to acknowledge that even within the protocol-adherence approach to training and practice, the importance of flexibility and adaptability is arguably still recognized (Kendall & Frank, 2018; Wiltsey Stirman et al., 2017). Many theorists, researchers, and clinicians appreciate that a one-size-fits-all approach to psychotherapy is limited, and even treatments with the most research support have the potential to result in negative outcomes for certain patients or under certain circumstances (Castonguay et al., 2010). Notably, results from a meta-analysis of studies that directly compared manualized versus non-manualized treatments failed to find significant outcome differences (Truijens et al., 2019), which generally supports there being benefit to therapist plasticity and clinical improvisation. Moreover, studies have demonstrated the potentially detrimental effects of *rigidly* adhering to a treatment protocol (e.g., Castonguay et al., 1996), as well as the potential benefits of within-adherence *flexibility* (i.e., the natural integration of techniques from other approaches; Owen & Hilsenroth, 2014).

Another sign of the growing recognition of the importance of flexibility and adaptability can be found in transdiagnostic (e.g., Barlow et al., 2017) and modular (e.g., Weisz & Chorpita, 2012) treatments, which explicitly instruct therapists to select from a menu of potential strategies and sequence them in different ways, and for different durations, from patient to patient. In addition, approaches to integrating model-exogenous strategies into CBT have been proposed and tested (e.g., Constantino et al., 2008). Such approaches are consistent with the emerging evidence base and are likely to be more consistent with how therapists operate in routine practice (Weisz & Chorpita, 2012). However, the field has been slow to adopt coinciding training methods.

An underlying feature of evidence-informed flexibility and adaptation is the meta-competency of responsiveness (Castonguay et al., 2023). Such action involves responding appropriately to the clinical context, both at the start of treatment (e.g., selecting the most suitable initial intervention) and during sessions in key moments (e.g., when a patient feels micro-aggressed against; Constantino et al., 2023). For clinical training and practice, both pre- and within-treatment responsiveness imply an *if-then* decision-making scheme (e.g., if a patient presents with these characteristics, then begin treatment with this CBT module; if a patient views a CBT intervention as low in credibility, then shift to a different CBT strategy or to a different therapy that has a more personally credible rationale). One training framework that privileges such if-then decision-making is *context-responsive psychotherapy integration* (CRPI; Constantino et al., 2013, 2023).

## Context-Responsive Psychotherapy Integration

To guide clinical training and practice, CRPI supports the use of timely evidence-based strategies that can be employed in response to the identification of specific and commonly occurring treatment markers (Constantino et al., 2020, 2023). These markers can include patient characteristics and within-session processes, which sometimes call for “staying the course” (e.g., when a current strategy is mutually agreed upon and achieving the expected or intended impact) or doing something deliberate and possibly different when particular contexts that have established relevance for patient outcomes present themselves. Such contextual markers have been, and can continue to be, identified through research and systemic clinical observations. For example, some clinical scholars have identified the following notable candidate “if” markers that may indicate a need to “then” engage in a clinical departure (either temporarily or more permanently): alliance ruptures, low patient motivation or change ambivalence, diminished patient outcome expectation, missed cultural opportunities or missteps, and not-on-track signals from routine outcomes monitoring (ROM) (Constantino et al., 2013, 2020; Constantino, Goodwin, et al., 2021; Constantino et al., 2023).

### Example Candidate Markers and Responses

#### Alliance Rupture-Repair

The quality of the therapeutic alliance is a well-recognized contributor to treatment outcome across different psychotherapies for various mental health concerns (Flückiger et al., 2018). Alliance ruptures reflect negative shifts in the patient-therapist bond or collaboration and are associated with maladaptive treatment processes and outcomes (Eubanks et al., 2018). Rupture markers are thought to typically fall into one or both of two categories: withdrawal or confrontation (both of which can be overt or covert). Thus, to be engaging in evidence-based practice beyond the aforementioned delivery of ESTs, therapists must be equipped to recognize potential rupture markers (*if*) and respond to them skillfully (*then*)—which may often require at least a temporary departure from the existing treatment plan (especially one that is not centered on interpersonal processes within the patient-therapist relationship). Theory and research point to some core resolution strategies, such as inviting patients to discuss potential problems in the relationship, exploring and validating patients’ experience of the rupture, and taking at least partial responsibility for the rupture (Constantino et al., 2008; Eubanks et al., 2018). These strategies are core components of *alliance-focused training* (Eubanks-Carter et al., 2015). Contrary to an earlier meta-analysis, Eubanks et al. (2018) did not find a statistically significant effect of rupture-resolution training on patient outcome. However, they examined theoretical model as a potential moderator of the training-outcome association and found that rupture-resolution training was associated with better patient outcomes in CBT-oriented treatments when compared to psychodynamic treatments.

#### Missed Cultural Opportunities or Missteps

Psychotherapy quality disparities exist for patients with underrepresented and historically marginalized sociocultural identities (e.g., race/ethnicity, sexual orientation, gender, economic, etc., McGuire & Miranda, 2008). Awareness of and responsiveness to such identities and associated contextual factors is part of evidence-based practice, yet research findings illuminate that patients often experience their therapist as missing the cultural or identity mark (Owen et al., 2016, 2018). Moreover, patients often view their therapist as engaging in potentially harmful behaviors and microaggressions (Hook et al., 2016). Consistent with the broader alliance rupture-repair literature, engaging in potentially harmful behavior (of omission or commission) in the absence of acknowledgment and steps to address it is worse for patient outcome than engaging in potentially harmful behavior and making an explicit attempt to address and correct it (Yeo & Torres-Harding, 2021). To help guide training and practice in making attempts to redress cultural missteps, the multicultural orientation framework outlines three transtheoretical and transdiagnostic therapist factors/actions: cultural comfort, cultural humility, and cultural opportunities (Davis et al., 2018). The latter stresses the ever-present importance of identifying (*if*) and responding to (*then*) cultural- and identity-relevant patient characteristics and communications in session. Such markers can be present when patients express a belief or value, discuss a role, or mention other personally relevant characteristics (e.g., family customs). Owen et al. (2016) found that patients who perceived a higher degree of missed cultural opportunities from their therapist also reported poorer treatment outcomes, yet this negative effect was attenuated when patients perceived the same therapist as possessing above average cultural humility. Though in need of further testing, personally tailoring treatment and responding to a patient’s salient (and especially marginalized) sociocultural identities holds promise for better addressing long-standing quality disparities in mental health care.

#### Routine Outcomes Monitoring

As another framework for guiding evidence-informed training and practice, ROM involves routinely assessing patient progress using standardized tools, and then integrating the feedback from these assessments into treatment decision-making. There is convincing evidence that the integration of ROM feedback into routine psychotherapy enhances patient improvement relative to routine care without ROM feedback (de Jong et al., 2021). Procedurally, many of the controlled ROM feedback-outcome studies have involved systems that alert a therapist when their patient is “not on track” (NOT) for an expected positive outcome based on predictive modeling. Notably, the magnitude of the ROM-feedback effect is further enhanced for these NOT cases. Thus, this negative outcome risk signal (*if*) can prompt the therapist to consider specific actions (*then*) to address the problem (e.g., learning that a patient has a recently diminished social support network) and get the psychotherapy back on track. Evidence indicates that a therapist’s subsequent attention to potentially relevant factors for NOT cases, such as alliance quality, social determinants, and treatment intensity, further reduces the risk of a negative outcome (Barkham et al., 2023). In fact, the relevance of routine monitoring to aid clinical responsiveness extends beyond outcome scales, with some systems monitoring and providing valued feedback on process variables, such as the working alliance and motivation (e.g., Demir et al., 2022). A focus on such relevant *if-then* scenarios may require unique and complementary training methods. Next, we identify and briefly discuss potential training structures and activities to support CRPI implementation.

## Training Activities

Although a comprehensive review of the CRPI framework and its implementation is beyond the scope of this article, we comment briefly on potential training structures and foci. Process research findings are the foundation of CRPI, including what is known about clinically relevant markers and the responsive clinical strategies that typically optimize outcomes. Taking alliance rupture-repair as an example, one must learn how to identify rupture markers and then repair them. We conceive of this *if-then* scenario as a potential training *module*. Psychotherapy courses and practica could be designed to cover a series of such modules (e.g., rupture identification and resolution training), which could be delivered in efficient doses that heighten their appeal to trainees. In addition, these training modules can be packaged in training videos for use by licensed professionals as part of continuing education. We now provide a few select examples of potential training activities.

### Training on First-Step Responsiveness

Addressing this first form of responsiveness, one key task is for trainees to become at least conversational on key principles and strategies from as many theoretical models as possible (Constantino et al., 2023). This breadth of theoretical and practical knowledge will allow therapists to maximize their ability to flexibly offer personalized therapy directions that a *given patient* finds credible and inspiring (e.g., one patient may find behavioral notions of exposure personally compelling, whereas another may find credible the idea of exploring relationship patterns about which they may be currently unaware). As another key task, trainees should become humbly knowledgeable about their own strengths and weaknesses (as grounded in patient outcomes data) in treating specific types of problem domains or using certain types of therapeutic interventions or processes. Doing so will both allow current personalization to the patient (by matching patients to therapists’ current strengths; Constantino, Boswell, et al., 2021) and personalization to the therapist (by directing training efforts to fortify strengths and improve weaknesses; Coyne et al., 2022).

### Training on Timely Departures in Response to In-Session Markers

Addressing this second form of responsiveness, one key task is for trainees to gain proficiency in marker identification. In addition to reading and hearing about markers, training therapists in process coding schemes is a complementary and potentially fruitful training activity that is receiving increased attention (Westra & Di Bartolomeo, in press). An example observational coding system is the Rupture Resolution Rating System (3RS; Eubanks et al., 2019), which identifies the presence of within-session alliance ruptures and therapist engagement in resolution strategies. The 3RS can help trainee-therapists learn how to identify rupture markers and their surrounding nuance, and demonstrate therapist resolution attempts, whether good, bad, or ugly. Viewing and discussing these behaviors is likely to be a useful training activity, yet it will ultimately need to be augmented to support its translation to trainees’ own clinical practice. To facilitate this translation, deliberate practice methods provide an opportunity for trainees to engage in repeated practice and to receive more direct feedback regarding marker identification and responsive behavior (Rousmaniere, 2017). The responsive behavior component reflects the second key task in training on timely clinical departures. For example, trainees can both learn and practice pointed theory-driven strategies that have an empirically demonstrated greater likelihood of effectively addressing contextual markers (such as using motivational interviewing to address diminished motivation for change), rather than simply adhering to the original treatment course (e.g., Westra et al., 2016, 2021).

## Recommendations and Future Directions

As the field reckons with growing evidence for therapist flexibility over strict model adherence, it stands to reason that research should place more weight on the therapist themselves and the benefit of their timely, in-the-moment interventions. It is notable that psychotherapy process research has uncovered more about potentially facilitative or hindering *patient* characteristics and behaviors than it has about *therapist* responses to these contexts (although see Ladmanová et al., 2022, for a qualitative meta-analysis of patient identified helpful events). For example, although therapist rupture repair, broadly conceived and measured, is associated with better outcome, we lack more precise empirical evidence supporting the effectiveness of specific resolution strategies for specific types of alliance rupture. A similar absence of fine-grained if-then empirical evidence exists to guide responsiveness to ROM markers, although innovations such as the Trier Treatment Navigator (TTN; Lutz et al., 2019) have shown promise to advance both marker detection and intervention selection. Furthermore, any shifts in our training models will also need to account for therapist differences in using interventions to beneficial effect, including with “then” responses to identifiable “if” markers; that is, even if everyone learned to notice a key marker, we need to contend with the fact that no one responsiveness strategy is likely to be effective in the hands of *all* clinicians. This complexity necessitates another thread of future research to match clinician to responsiveness *options* in order to optimally address negative process.

Similar challenges can be found on the patient side. For example, in its fullest form, context-responsiveness is concerned with responding to both patient characteristics (e.g., initial treatment selection) and within-treatment markers (e.g., alliance ruptures). It would reinforce uniformity myths in psychotherapy to assume that a particular therapist response will be optimal for all patients with a particular pre-treatment characteristic or all instances of a particular within-session process marker. Accordingly, optimizing our training paradigm to align with the realities of real-world practice will require that psychotherapy process research continue to explicitly uncover a range of key contextual markers (for a precedent of such work, see Greenberg & Watson, 2006) and test the best ways therapists can respond to them.

In addition, there is both direct and indirect evidence that even clinically and empirically well-grounded training interventions can be associated with negative consequences in specific contexts (Castonguay et al., 2010). Furthermore, the universal trainability of process-acuity related skills, in particular, remains an open question. Individual differences among therapists present a key challenge to training implementation. Training methods, such as deliberate practice, emphasize the importance of tailoring the focus and difficulty of training to the individual (Rousmaniere, 2017), yet more research on deliberate practice implementation process and outcome is required.

The need for more research on training implementation process and outcome is not unique to deliberate practice and remains broadly relevant to pre- and post-graduate training in psychotherapy (Knox & Hill, 2021). Although the benefit of context-responsiveness integration has been demonstrated in multiple research studies (e.g., Constantino et al., 2008; Westra et al., 2016), the feasibility of implementing the CRPI framework in routine practice and training contexts remains an open question. As described at the beginning of this article, the prevailing training structures and philosophy represent potential barriers. However, frameworks such as the CRPI embrace the inherent complexity and nuance of psychotherapy. Although rather speculative, this acknowledgement of complexity may ring truer for therapists, and thus, predict greater openness to adoption. Moreover, CRPI may have inherent adoption appeal in that the use of focused, timely, and often temporary “then” responses, which can be integrated into any foundational treatment being administered, does not require a clinician to dramatically change their professional identity—other than to include that they are an empirically responsive [insert treatment modality] clinician!
